# Decoding Complexity: Readability Analysis of Public Online Health Information on Common Neurological Diagnoses

**DOI:** 10.1002/alz.084394

**Published:** 2025-01-09

**Authors:** Manvi Punukollu, Archana Venkatesan

**Affiliations:** ^1^ Medical College of Georgia, Augusta, GA USA

## Abstract

**Background:**

Alzheimer’s disease poses significant global health challenges, particularly as people increasingly turn to online platforms for health information in the digital era. Given the intricate nature of Alzheimer’s, it is imperative to evaluate the readability of online content. This study aims to assess the readability of information related to Alzheimer’s disease on reputable health websites.

**Method:**

We carefully selected websites based on authority and prevalence through Google, the most popular search engine. Utilizing the neutral term “Alzheimer’s” ensured a broader sample of websites. We identified common domains within the top ten search results and meticulously sorted and saved them. Texts were then extracted and cleaned using Microsoft Office Excel. To evaluate readability, we employed various readability formulas using Python. The Flesch Reading Ease Score (FRES), Flesch–Kincaid grade level (FKGL), Simple Measure of Gobbledygook (SMOG), Gunning Fog (GFOG), Coleman–Liau score (CL), automated readability index (ARI), and Linsear Write (LW) were utilized.

**Result:**

When comparing our readability results to the sixth‐grade level recommended by the American Medical Association and the National Institute of Health, we observed that the scores exceeded the recommended level. Findings from widely used internet resources revealed variations in readability scores across different websites. Notably, publicly accessible data related to Alzheimer’s disease tends to be presented at a higher reading level than recommended for general comprehension, carrying critical implications for patient education and health literacy within the Alzheimer’s context.

**Conclusion:**

Assessing the readability of online health information for Alzheimer’s disease is crucial to ensure accessibility and comprehension for diverse audiences. This study provides valuable insights into the readability landscape of reputable health websites, uncovering a potential gap between the complexity of information and the general public’s reading ability in the context of Alzheimer’s disease. Recognizing and addressing this gap is essential for enhancing patient education and empowerment, ultimately contributing to improved health outcomes in the realm of Alzheimer’s.

